# Epigenetic control of the plant metabolome: implications for Chinese medicine

**DOI:** 10.1186/s13020-025-01217-8

**Published:** 2025-10-06

**Authors:** Tae Kyung Hyun

**Affiliations:** Department of Industrial Plant Science and Technology, College of Agriculture, Life and Environment Sciences, Chungbuk National University, Cheongju, 28644 Republic of Korea

**Keywords:** Epigenetic regulation, DNA methylation, Histone modification, Small RNAs, Specialized metabolism

## Abstract

Epigenetic regulation—including DNA methylation, histone modifications, and small RNAs—plays a crucial role in modulating specialized metabolism in plants; specialized metabolites confer crucial pharmacological properties to plants, forming the basis of traditional Chinese medicine (TCM). Recent studies have highlighted the influence of these mechanisms on metabolite production in key medicinal plants such as *Papaver somniferum* and *Salvia miltiorrhiza*. Emerging technologies such as CRISPR/dCas9-based epigenome editing and miRNA modulation enable precise control of biosynthetic pathways. Integrating these molecular-level strategies into TCM research and the cultivation of medicinal plants could improve the efficacy and consistency of herbal medicinal products, providing molecular tools for the targeted regulation of active ingredients and thereby facilitating the modernization of TCM.

## Background

**Fig. 1:**
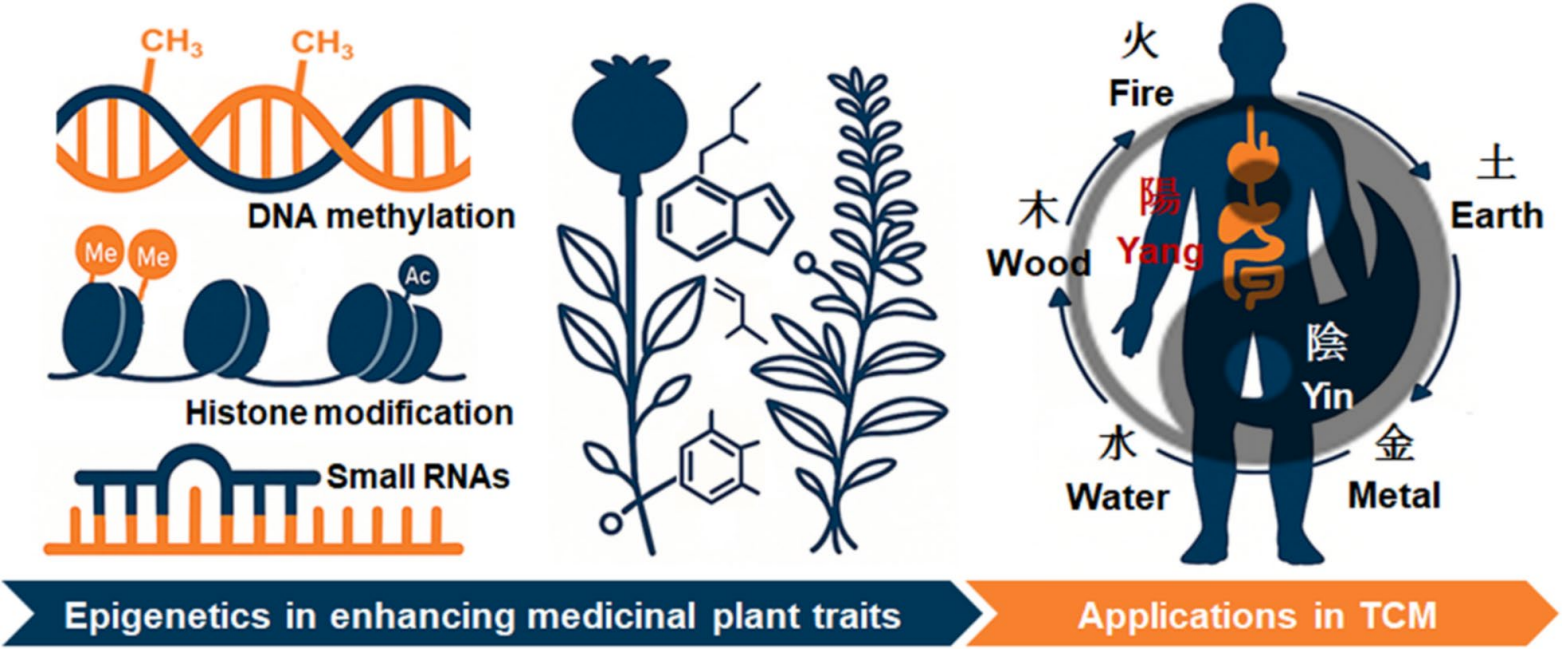
Epigenetic regulation of specialized metabolites in medicinal plants and its relevance to traditional Chinese medicine

Plants synthesize an immense variety of metabolites that are essential for their development, ecological adaptation, and defense. Among these, some specialized (also called secondary) metabolites are of particular importance to traditional Chinese medicine (TCM) as they constitute the pharmacological backbone of herbal formulations. While it is well established that the expression of these compounds is regulated at transcriptional and post-transcriptional levels, recent studies have shown that epigenetic regulation can further modulate these metabolic pathways by altering the gene expression environment, highlighting a critical yet underexplored role in shaping their biosynthesis [1, 2]. This commentary highlights recent advances in plant epigenetics and explores how mechanisms such as DNA methylation, histone modification, and small RNAs modulate phytochemical output, offering new avenues to enhance the efficacy, standardization, and modernization of TCM (Fig. 1).

## Mechanistic insights into the epigenetic control of specialized metabolism

Epigenetic processes—heritable yet reversible modifications that alter gene expression without changing the DNA sequence—are key regulators of plant metabolism. DNA methylation, histone modifications (e.g., methylation and acetylation), and small RNAs such as small interfering RNA (siRNA) and micro RNAs (miRNAs) orchestrate the transcriptional accessibility of biosynthetic genes [3]. Of these mechanisms, DNA methylation has been extensively studied for its impact on metabolite accumulation. One of the best-known examples is that of the *colorless nonripening* (*Cnr*) epiallele in tomato, wherein the hypermethylation of the promotor region of a gene encoding a ripening-related transcription factor results in reduced carotenoid accumulation in the fruit [4]. Similarly, a naturally occurring epiallele, *Vitamin E 3* (*VTE3*), downregulates tocopherol biosynthesis via siRNA–mediated DNA methylation, in which siRNAs guide the methylation machinery to the VTE3 promoter, resulting in transcriptional silencing [5]. In *Papaver somniferum*, variation in DNA methylation status affects alkaloid content, underscoring the regulatory role of the epigenome in the production of specialized metabolites that possess medicinal properties [6]. Another case in point is *Salvia miltiorrhiza*, an herb widely used in TCM, in which DNA methylation affects the expression of several crucial enzymes for the biosynthesis of tanshinones and phenolic acids, including copalyl diphosphate synthase 5 (SmCPS5), cytochrome P450-related enzyme (SmCYP71D464), geranylgeranyl diphosphate synthase (SmGGPPS1), geranyl diphosphate synthase (SmGPPS), hydroxyphenylpyruvate reductase (SmHPPR), and hydroxyphenylpyruvate dioxygenase (SmHPPD); altered DNA methylation levels of these genes have been shown to correlate with significant changes in the accumulation of tanshinones and phenolic acids, such as rosmarinic acid and salvianolic acid B [7]. Like DNA methylation, histone modifications also contribute to metabolic regulation, although not all are directly involved in specialized metabolism. For example, in rice, the histone demethylase JMJ705, by removing the repressive H3K27me3 mark, enables the methyl jasmonate (MeJA)–responsive epigenetic activation of the diterpenoid gene cluster on chromosome 7—a cluster that drives the biosynthesis of casbene-type phytoalexins [8]. In poplar, JMJ25 regulates MYB transcription factor expression by demethylating lysine 9 of histone 3 (H3), thus suppressing anthocyanin biosynthesis [9]. In ginseng, histone deacetylase inhibitors enhance MeJA-induced ginsenoside biosynthesis by promoting H3K18 acetylation, thereby upregulating key biosynthetic genes while also indicating that histone deacetylases function as repressors of this biosynthetic pathway [10]. Aside from DNA methylation and histone modifications, miRNAs have emerged as critical post-transcriptional regulators in specialized metabolism, influencing the synthesis of specialized metabolites such as flavonoids, terpenoids, and alkaloids. For example, in sunflower, miR-2911 targets γ-tocopherol methyltransferase, thus regulating tocopherol synthesis. On the other hand, in clary, miR-828a and miR-948a enhance flavonoid biosynthesis by controlling *MYB12* and lipoxygenase expression [11]. Beyond their individual roles, epigenetic mechanisms often act synergistically. For instance, in *Brassica napus*, hypermethylation in the promoter region of *BnaCnng64040D* (lipase family protein), via miR-159-directed DNA methylation, resulted in a marked reduction of gene expression in flower buds [12]. Such multilayered regulation is likely to operate in medicinal plants as well and may fine-tune specialized metabolism. Together, these examples illustrate how multiple epigenetic layers converge to modulate specialized metabolism in plants. The dynamic interplay among DNA methylation, histone modifications, and small RNAs contributes to the fine-tuning of phytochemical output, enabling plants to respond to environmental cues and developmental signals with biochemical precision.

## Implications for Traditional Chinese medicine

Despite these breakthroughs, the integration of epigenetic insights into TCM research and herbal medicine production remains limited. As the global demand for TCM products—and with it the need for consistent quality and efficacy—grows epigenetic tools present an unprecedented opportunity. Emerging tools such as CRISPR/dCas9-based epigenome editing, histone modification modulators, DNA methylation regulators, and miRNA mimics or inhibitors can be employed to precisely control biosynthetic gene expression (Fig. 1). Although the use of CRISPR/dCas9-based systems to modify epigenetic marks in plants has been explored in only a few studies, evidence shows that targeting H3K27me3 at the Arabidopsis CUP SHAPED COTYLEDON 3 boundary gene can induce ectopic transcription and influence plant development [13]. These observations indicate that CRISPR/dCas9-mediated epigenome editing may allow precise spatial and temporal regulation of gene expression [14], providing the potential to selectively activate or repress specific metabolic pathways. In the future, such strategies could be utilized to enhance the production of valuable secondary metabolites, including artemisinin, baicalin, or morphine alkaloids, without altering the underlying DNA sequence. Moreover, understanding the impact of environmental factors on epigenetic mechanisms and, therefore, on metabolite profiles can help optimize cultivation and harvesting strategies for medicinal plants. Integrating principles of epigenetics into agriculture of medicinal plants used in TCM could help optimize phytochemical yields under field conditions, improving both therapeutic efficacy and resource sustainability. Such strategies align closely with national and international efforts to modernize Chinese medicine through evidence-based approaches and molecular precision.

## Conclusion

Epigenetic regulation presents a powerful, yet underutilized, dimension of plant specialized metabolism that holds direct relevance for TCM. Unlike previous reviews that address either plant epigenetics or TCM separately, this article systematically integrates these fields, highlighting how DNA methylation, histone modifications, and small RNAs collectively influence medicinal plant metabolites. Incorporating strategies of epigenetics into TCM herb development, quality control, and pharmacological assessment will not only enhance consistency and therapeutic reliability but may also reveal new bioactive potentials hidden within traditional remedies. Harnessing the plant epigenome with precision and purpose may become pivotal to the evolution of Chinese medicine from an empirical tradition to an evidence-based medical discipline.

## Data Availability

No datasets were generated or analysed during the current study.

## Abbreviations

Cnr: Colorless nonripening
CPS5: Copalyl diphosphate synthase 5
GGPPS: Geranylgeranyl diphosphate synthase
GPPS: Geranyl diphosphate synthase
H3: Histone 3
HPPD: Hydroxyphenylpyruvate dioxygenase
HPPR: Hydroxyphenylpyruvate reductase
MeJA: Methyl jasmonate
miRNA: Micro RNA
siRNA: Small interfering RNA
TCM: Traditional Chinese medicine
VTE3: Vitamin E 3

## Declarations

### Declaration of AI and AI-assisted technologies in manuscript preparation processes

During the preparation of this manuscript, the author utilized ChatGPT for language refinement.
